# Supplementary material for: U.S. household food acquisition behaviors during the COVID-19 pandemic
Source: PLoS One. 2022 Jul 14;17(7):e0271522. doi: 10.1371/journal.pone.0271522 (PMC9282540; doi:10.1371/journal.pone.0271522)
Supplement: Attachment — Submitted filename: Response to Reviewers 052622.docx [file pone.0271522.s002.docx]

*Thank you for the helpful comments on the paper. We have revised the paper in response to the comments and hope you find the paper acceptable for publication. Below are the original comments which are followed by our responses in italics.*

Reviewer #1: Authors aim to analyze eight food acquisition activities (in person grocery shopping; online grocery shopping; ordering from a meal kit service; indoor restaurant dining; outdoor restaurant dining; ordering take-out from a restaurant; visiting a food bank; and visiting a farmer’s market) at different points in the pandemic, which allows them to evaluate how food acquisition changed as case rates changed and vaccine rollouts occurred.

Abstract

OK. However, conclusions are not completely clear for me. I can see many results, but not conclusions. Authors should clearly highlight their conclusions.

*Thank you for this suggestion. We have streamlined the abstract to more succinctly present the results and outline our key conclusions (see lines 16-33).*

Introduction.

Authors properly settled the context and pointed out their aims as well as the study’s contributions.

*Thank you for the positive feedback on the introduction.*

Methods

They collected data from three nationally representative online samples in September 2020, December 2020, and March 2021. In each cross-sectional wave of the survey, they recruited approximately 1000 U.S. consumers to complete an online survey in the Qualtrics survey platform.

They did not recruit the same individuals to participate in each wave of the survey; each sample was recruited to be representative of the U.S. population in terms of gender, age, income, and geographic region. Individuals were eligible to participate if they were 18 years of age or older and responsible for at least 50% of the grocery shopping in their household.

Survey used a very short form to analyze how households acquired foo throughout the COVID-19 pandemic. Participants were also asked to complete a series of sociodemographic questions, including questions related to gender, age, income, education, race, geographic, metropolitan status, political affiliation, and nutrition assistance recipient status.

Inclusion and exclusion criteria were defined. Finally, they evaluated changes across time and across demographics using a multivariate probit model.

*We appreciate the reviewer’s summary of our work.*

Results

OK

Discussion/Conclusion

Although not mandatory, this section should be splitting into Discussion and Conclusions. This could be related with the problem I found into the Abstract. Conclusions can be drafted as a take-home message.

*Thank you for this suggestion. While the journal allows for a combined discussion/conclusion section, we appreciate the reviewer’s feedback to differentiate our conclusion from the discussion of results. To that end, we have added a separate conclusion section (see lines 361-375) that details two key findings: 1) the potential complementarities between in-person and online grocery shopping and 2) restaurants’ vulnerability to COVID-19 surges (relative to grocery retailers). We present implications for grocery stores and restaurants related to these findings as they continue to navigate operational challenges associated with the COVID-19 pandemic. We hope these changes have helped to clarify our conclusions.*

Reviewer #2: I would like to congratulate the authors for this study, it is very interesting. This study shows the more frequent food acquisition activities from U.S consumers during different periods of time of the COVID-19 pandemic. So, the manuscript has been written appropriately, providing literature related to the topic.

*Thank you for the positive feedback on our study – we appreciate you taking the time to review it!*
